# Vaccine development against Schmallenberg virus: from classical inactivated to modified-live to scaffold particle vaccines

**DOI:** 10.1186/s42522-022-00069-8

**Published:** 2022-08-17

**Authors:** Kerstin Wernike, Andrea Aebischer, Jean-Christophe Audonnet, Martin Beer

**Affiliations:** 1 Friedrich-Loeffler-Institut, Südufer 10, 17493 Greifswald – Insel Riems, Germany; 2 Coordinator of ZAPI IMI Project, Retired from Boehringer Ingelheim Animal Health, Lyon, France

**Keywords:** *Bunyavirales*, Peribunyavirus, Orthobunyavirus, Vaccination

## Abstract

**Background:**

Subsequent to its first detection in 2011, the insect-transmitted bunyavirus Schmallenberg virus (SBV; genus *Orthobunyavirus*) caused a large-scale epizootic of fetal malformation in the European ruminant population. By now, SBV established an enzootic status in Central Europe with regular wave-like re-emergence, which has prompted intensive research efforts in order to elucidate the pathogenesis and to develop countermeasures. Since different orthobunyaviruses share a very similar structural organization, SBV has become an important model virus to study orthobunyaviruses in general and for the development of vaccines. In this review article, we summarize which vaccine formulations have been tested to prevent SBV infections in livestock animals.

**Main:**

In a first step, inactivated SBV candidate vaccines were developed, which efficiently protected against an experimental SBV infection. Due to the inability to differentiate infected from vaccinated animals (= DIVA capability), a series of further approaches ranging from modified live, live-vectored, subunit and DNA-mediated vaccine delivery to multimeric antigen-presentation on scaffold particles was developed and evaluated. In short, it was repeatedly demonstrated that the N-terminal half of the glycoprotein Gc, composed of the Gc head and the head-stalk, is highly immunogenic, with a superior immunogenicity of the complete head-stalk domain compared to the Gc head only. Furthermore, in all Gc protein-based vaccine candidates, immunized animals can be readily discriminated from animals infected with the field virus by the absence of antibodies against the viral N-protein.

**Conclusions:**

Using SBV as a model virus, several vaccination-challenge studies in target species underscored the superior performance of antigenic domains compared to linear epitopes regarding their immunogenicity. In addition, it could be shown that holistic approaches combining immunization-challenge infection studies with structural analyses provide essential knowledge required for an improved vaccine design.

## Background

In contrast to coronaviruses, which are widely known nowadays, bunyaviruses are less well-known, although they represent one of the largest group of viruses. The order *Bunyavirales* consists of 46 genera assigned to 12 families and accommodates thousands of viruses with linear, segmented, single-stranded RNA genome [1, 2]. One of the genera that belongs to the family *Peribunyaviridae* is the *Orthobunyavirus* genus, which contains more than 170 insect-transmitted viruses assigned to 18 serogroups. Of these, the Simbu serogroup is not only one of the largest, but also the most important group in terms of veterinary public health, since it contains e.g. Schmallenberg virus (SBV), Akabane virus (AKAV) and Shuni virus (SHUV) [3].

In general, viruses of the genus *Orthobunyavirus* are spherical, about 100 nm in diameter and relatively simple in their composition. The tripartite single-stranded RNA genome encodes for only four structural and two non-structural proteins. The large (L) genomic segment encodes for the RNA-dependent RNA polymerase, while the medium (M) segment encodes for the surface glycoproteins Gn and Gc and the non-structural protein NSm. Gn and Gc are integral transmembrane proteins, which play an important role in viral attachment, membrane fusion and the induction of the host’s immune response [4–6]. The small (S) segment encodes for the nucleocapsid protein N and the non-structural protein NSs, which acts as interferon antagonist and represents a major virulence factor in vertebrate hosts [7–9]. The N-protein forms ribonucleoprotein complexes with the three viral RNA segments that are further associated with the L-protein, and this “super complex” is essential for viral RNA transcription and replication [8, 9].

An important representative of the *Orthobunyavirus* genus is SBV. The virus was detected for the first time in late 2011 at the German-Dutch border region [10]. Thereafter, it rapidly spread throughout the European continent and established an enzootic status in Central Europe [11, 12]. SBV is transmitted by *Culicoides* biting midges and infects predominantly ruminants [3]. Infections of adult animals are either asymptomatic or associated with mild, unspecific clinical signs such as fever, diarrhea and decrease in milk yield for a few days [10, 13]. Infections of naïve dams during a critical phase of gestation, however, may result in abortion, premature birth, stillbirth or the birth of severely malformed lambs and calves. The malformations affect the central nervous and musculoskeletal system and are summarized under the term arthrogryposis-hydranencephaly syndrome [13]. Since different orthobunyaviruses share a very similar structural organization, SBV has become an important model virus to study these viruses in general, and for the development of vaccines and therapeutic reagents to combat new infectious diseases and potentially zoonotic threats.

Since treatment options are not available against the insect-transmitted orthobunyaviruses, further possibilities need to be discussed to protect hosts from clinical disease. From an entomological point of view, the application of repellents or insecticides could be considered in order to prevent infected vectors from biting susceptible animals. Furthermore, measures controlling vector development, like e.g. environmental interventions to remove larval breeding sites, could be taken into consideration. In addition, naïve females could be housed in insect-proof buildings. However, most of these measures seem impractical, cumbersome, expensive and have only a very limited or a not significant effect [11, 14]. Hence, vector-transmitted orthobunyaviruses are difficult to control by vector management only. In contrast, European experiences during the outbreak of the likewise *Culicoides*-transmitted, ruminant infecting bluetongue virus from 2006 to 2009 have demonstrated that vaccination campaigns against a *Culicoides*-borne disease can be a very effective tool for disease prevention or even for eradicating a disease from a given area [15, 16]. Based on this knowledge, SBV-specific vaccines have been developed within a short timeframe. Here, we describe the vaccine formulations that have been tested to prevent SBV infections and discuss the rationale underlying the application of each platform.

## Main text

### Classical inactivated vaccines as an early response measure

**Fig. 1:**
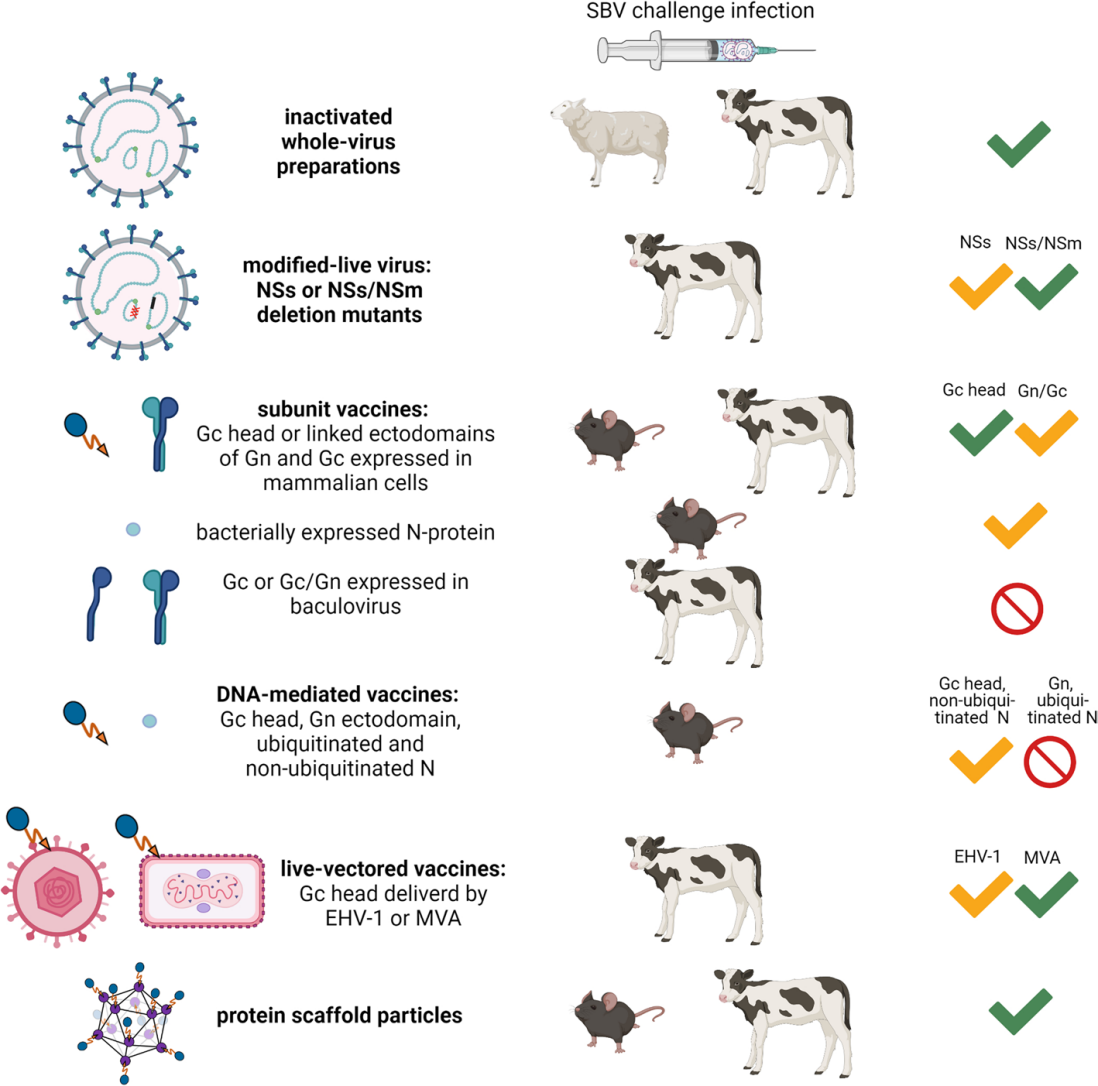
Types of vaccines developed against infections with Schmallenberg virus (SBV). The animal species, in which each type of vaccine was tested, is shown. Green check marks symbolize complete protection and yellow check marks partial protection. Created with BioRender.com

A common, rapid and relatively inexpensive approach for vaccine development is the chemical inactivation of whole-virus preparations. Indeed, such inactivated vaccine formulations exist for viruses closely related to SBV. A Japanese multivalent vaccine against the simbuviruses AKAV, Aino virus (AINOV) and the similarly teratogenic reovirus, Chuzan virus, is applied to prevent reproductive disorders in ruminants [17]. At the time of the first detection of SBV, specific vaccines were of course not yet available. Provided its efficacy against SBV infection, this already existing vaccine, developed against closely related viruses, would have been a fast and convenient tool for SBV disease control. Unfortunately, the heterologous trivalent vaccine did not confer protection against an infectious SBV challenge [18]. Hence, homologous effective vaccines had to be produced as fast as possible to prevent clinical disease and further virus spread. Several SBV-specific inactivated vaccines were indeed developed and tested regarding their protective efficacy in cattle and sheep after two immunizations [19]. In addition, the efficacy of one of these preparations was tested in sheep after a single immunization, as a reduction to a single injection may minimize costs and workload and could ensure a more rapid immunization of the target animal population in a given area. This is especially important for sheep owners, since the animals have to be individually caught and restrained on the pastures for every injection. In short, four out of five inactivated SBV candidate vaccines completely prevented RNAemia, the fifth reduced it considerably [19], and sheep were even protected against an experimental SBV infection after a single vaccination [20] (Fig. 1). By now, marketing authorizations have been granted for three inactivated whole-virus vaccines. These vaccines were licensed for the British and French market in 2013, and in May 2015, such a vaccine received a European Union central marketing authorization [21–23].

### On the way to DIVA-compatible vaccines: modified live, subunit, DNA-mediated and vectored vaccines

Though highly effective in preventing viremia, the whole-virus inactivated vaccines have a major drawback: they do not enable the differentiation of infected from vaccinated animals (DIVA). However, DIVA-capable vaccines would offer several advantages, first of all the possibility to demonstrate freedom of disease by serological methods after an outbreak or to grant the safe movement of susceptible animals between affected and disease-free countries. For Rift Valley fever virus (RVFV), a mosquito-transmitted phlebovirus from the *Bunyavirales* order, DIVA-capable vaccine candidates have previously been developed and insights in these experimental data were used as a basis to develop similar SBV-specific vaccines. On one hand, RVFV mutant viruses lacking non-structural proteins NSs and NSm were shown to replicate efficiently in cell culture and, besides being DIVA-compatible, they protected rats and sheep from viremia after experimental RVFV infection [24, 25]. On the other hand, a RVFV subunit vaccine based on the viral glycoproteins Gn and Gc conferred sterile protection of sheep against virulent virus challenge [26].

When taking these approaches as a basis for the development of SBV-specific vaccines, one should keep in mind that the construction, viability and vaccine suitability of SBV mutants with deletions of NSs and NSm may be complicated by the differing coding strategies of orthobunyaviruses and phleboviruses. While the small genomic segment (S) of orthobunyaviruses encodes for the nucleocapsid protein N and for the non-structural protein NSs in alternative overlapping reading frames [27], the S-segment of phleboviruses uses an ambisense coding strategy, encoding the N protein in the negative-sense orientation, and the NSs protein in the positive-sense [28]. The M-segment of bunyaviruses encodes for a precursor protein, which is post-translationally processed into the two glycoproteins Gn and Gc and, with the exception of hantaviruses, the non-structural protein NSm. However, the genome organization differs between the genera. While the orthobunyaviral NSm protein is encoded between the Gn and Gc glycoproteins, the coding order of the M-segment of phleboviruses is NSm-Gn-Gc [27], making the NSm-encoding region more readily accessible for manipulation.

Nevertheless, SBV NSs-, NSm- and combined NSs/NSm-double deletion mutants could be successfully generated and were tested for their ability to protect cattle from SBV challenge infection [29]. Three out of four cattle immunized once with the NSs-deletion mutant and all animals vaccinated with the virus lacking both nonstructural proteins were fully protected against challenge infection (Fig. 1). Unfortunately, diagnostic test systems allowing differentiation of infected animals from individuals immunized with one of these modified-live vaccines are still missing [29].

In addition to the modified-live vaccines, candidate subunit vaccines have been described for RVFV [26] and they likewise served as a model for the construction of SBV-specific subunit and vector-based vaccines. However, prior to the selection of a suitable delivery system, antigens had to be identified that could stimulate a protective immune response in infected hosts. Domains that are targeted by neutralizing antibodies were identified within the SBV Gc glycoprotein by analyzing the reactivity of a set of monoclonal antibodies and antisera [30]. Sera from convalescent animals reacted only against the full-length Gc-protein and its subdomains, and not against the glycoprotein Gn [30]. Based on these analyses, the N-terminal domain of Gc (termed Gc head) was considered to be a suitable immunogen for DNA-mediated, vector-based or subunit vaccines. Indeed, the potential of the Gc head domain as a promising vaccine candidate could be confirmed in several vaccination-challenge trials, both in a small animal model (type I interferon receptor knock-out (IFNAR-/-) mice) and/or in cattle [31–33]. It was also shown that its immunogenicity highly depends on a correct conformation of the antigen. For vector-based vaccines, the replication of the vector virus in the vaccinated animals additionally plays a crucial role. Immunization with Gc head-encoding DNA plasmids and recombinant Gc head expressed in a mammalian expression system conferred protection to a certain limit in a subset of animals [31, 34]. An excellent performance was eventually achieved using the genetically fused Gc head domains of SBV and the related AKAV, which fully protected vaccinated mice and cattle against SBV challenge infection. In contrast, neither the bacterially expressed nor the reduced form of Gc head protected from SBV infection. The same was observed for a full-length Gc construct expressed in baculovirus [31, 35].

In order to gain insights in the molecular architecture of the SBV spike protein, X-ray crystallography studies were performed and it could be shown that the N-terminal half of Gc is composed of a head and a stalk domain. Furthermore, it was demonstrated, that the larger Gc head-stalk domain (aa 465–874) had an even higher efficacy than the Gc head (aa 465–702) only. Vaccination with this construct conferred a virtually sterile immunity in mice [33].

For the design of Gc-based SBV-specific vector vaccines, two viral vectors previously described for vaccine development against other cattle diseases have been selected, namely equine herpesvirus type 1 (EHV-1) and the Modified Vaccinia Ankara (MVA) poxvirus. While the EHV-1-based formulation conferred protection in two out of four immunized cattle, vaccination with the MVA-based vector vaccine efficiently induced an SBV-specific antibody response and fully protected the vaccinated animals against challenge infection [32].

In all those Gc-based approaches, vaccinated animals could be readily discriminated from animals infected with the field virus by the absence of antibodies against the SBV N-protein in immunized animals [31–33].

As an alternative target for the development of DNA-mediated or subunit vaccines, the viral N-protein has been suggested [34, 36]. Using the small animal IFNAR-/- mouse model, it could be shown that bacterially expressed or DNA-mediated N could confer a certain degree of protection, as the level of viremia was reduced and the clinical signs decreased in immunized mice of some groups [34, 36]. However, the efficiency testing in ruminants, i.e. the target species of SBV, is still pending.

### Protein scaffold particles: a novel platform for multivalent antigen display

In a very recent study, a modular vaccine platform for multimeric antigen display has been developed based on the lumazine synthase (LS) from *Aquifex aeolicus* as a scaffold protein (multimeric protein scaffold particle, MPSP). Using SBV as a model, it could be shown that the LS allows the presentation of genetically fused linear peptide epitopes as well as of complex antigenic domains using a bacterial superglue plug-and-display strategy [37].

Since previous vaccine trials repeatedly demonstrated the immunogenicity of the N-terminal half of the SBV Gc [31–33], the head (aa 465–702) as well as the head-stalk (aa 465–874) domain were selected as model antigens to evaluate the functionality and applicability of the newly developed vaccine platform. For this purpose, the recombinant proteins were expressed in insect *Drosophila S2* cells (Gc head) or in the C1 fungal system (Gc head-stalk) and subsequently conjugated to the pre-fabricated LS-MPSP by spontaneous isopeptide formation [37]. A linear peptide epitope located at the interface of the Gc head and stalk domain (aa 694–708) was identified by Pepscan analysis and applied to test a genetic fusion approach in parallel. Using the selected model antigens, LS-MPSP-based vaccine candidates were designed and their immunogenicity was evaluated in a small animal model as well as in cattle, a major target species of SBV [37]. In the IFNAR-/- mouse model it could be shown, that a multimeric presentation of the Gc head domain on LS-MPSPs markedly improved vaccine efficacy compared to the monomeric subunit. A partial protection from an otherwise lethal challenge infection could be achieved even after a single shot vaccination. The C1-produced Gc head-stalk antigen induced a potent immune response and conjugated to LS-MPSPs it conferred complete clinical protection with or without a booster immunization, confirming the adjuvanting effect of the antigen display on MPSPs. This data further confirmed the previously reported superior immunogenicity of the head-stalk domain compared to the Gc head only [33].

The efficacy of LS-MPSPs displaying the recombinant proteins or the selected linear peptide epitope was also assessed in cattle, a target species of SBV. After prime-boost immunization, MPSPs displaying the Gc head or head-stalk domain induced high titers of neutralizing antibodies in all vaccinated animals and conferred a sterile immunity. Despite a good performance in the IFNAR-/- mouse model, the peptide vaccine failed to induce a protective immune response in cattle [37]. This underscores the superior performance of antigenic domains compared to linear epitopes with regard to immunogenicity, and further shows the value of a functional target species model to validate vaccine efficacy early on in development.

## Conclusions and lessons learned for rational vaccine design

Emerging and re-emerging infectious diseases nowadays represent a major threat for human and animal health. In order to allow a quick response when novel pathogens emerge, continued innovation in designing and studying efficacious vaccines is needed. SBV has become a suitable model for a newly emerging virus. Since functional small animal and target animal models are readily available, SBV was repeatedly applied for vaccination-challenge studies to evaluate the potential of novel vaccine technologies. Apart from several conventional approaches, a modular platform allowing the presentation of complex antigenic domains on multimeric protein scaffolds using a bacterial superglue plug-and-display strategy was developed using SBV as a model. This platform proved to be a promising tool for the design and production of suitable vaccines as soon as a major immunogenic domain of a new pathogen has been identified. Furthermore, SBV vaccine studies demonstrated that holistic approaches combining immunogenicity studies with structural analyses provide essential knowledge required for promising vaccine design.

Hence, the history of SBV vaccine development and especially the use of modern technologies like multimeric protein scaffolds could be a blueprint for a general workflow, which consists of the selection of major immunogens for a given pathogen by combining the analysis of the 3D structure and the identification of the most immunogenic sub-domains using sera of naturally or experimentally infected individuals as a prerequisite, followed by the production of the selected key immunogens in suitable expression and presentation systems and finally, in vivo studies in model hosts and target species to assess the immunogenicity and efficacy performances.

## Data Availability

Not applicable.

## Abbreviations

AINOV: Aino virus
AKAV: Akabane virus
DIVA: Differentiation of infected from vaccinated animals
EHV-1: Equine herpesvirus type 1
IFNAR-/-: Type I interferon receptor knock-out
LS: Lumazine synthase
MPSP: Multimeric protein scaffold particle
MVA: Modified vaccinia virus Ankara
RVFV: Rift Valley fever virus
SBV: Schmallenberg virus
SHUV: Shuni virus
